# Supplementary material for: Free Radical Copolymerization of N-Isopropylacrylamide and 2,3-Dihydroxypropyl Methacrylate: Reaction Kinetics and Characterizations
Source: Materials (Basel). 2025 Apr 2;18(7):1614. doi: 10.3390/ma18071614 (PMC11990630; doi:10.3390/ma18071614)
Supplement: Supplementary file 1 [file materials-18-01614-s001.zip › materials-3549603-supplementary.pdf]

# Free radical copolymerization of *N*-isopropylacrylamide and 2,3-dihydroxypropyl methacrylate: Reaction kinetics and characterizations

**Zhishu Chen [1,2] and Chao Zhang [1, 2, *]**

[1] *School of Biomedical Engineering, Shenzhen Campus of Sun Yat-sen University, Shenzhen, Guangdong, 518107, China.*
[2] *Guangdong Provincial Key Laboratory of Sensor Technology and Biomedical Instrument, Sun Yat-sen University, Shenzhen, Guangdong, 518107, China.*
[*] *Correspondence: zhchao9@mail.sysu.edu.cn*

## 1. Results

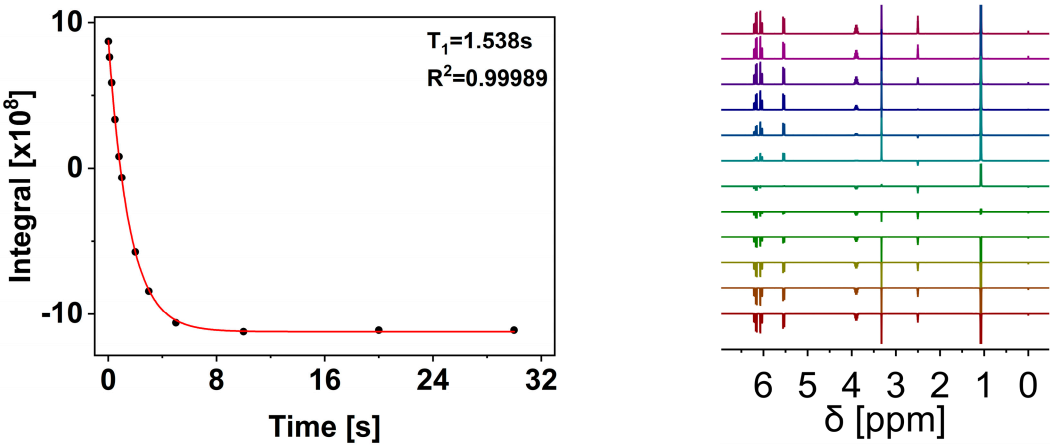


(a)

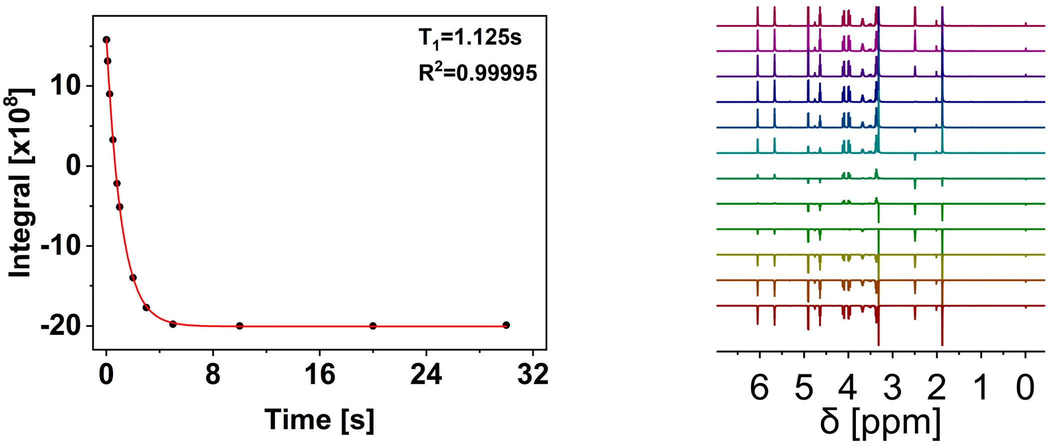


(b)

**Figure S1.** $T_1$ (Left) and the $^1$H NMR that was used for fitting (Right). (a) NIPAm (δ=5.54 ppm) (b) DHPMA (δ=5.67 ppm).

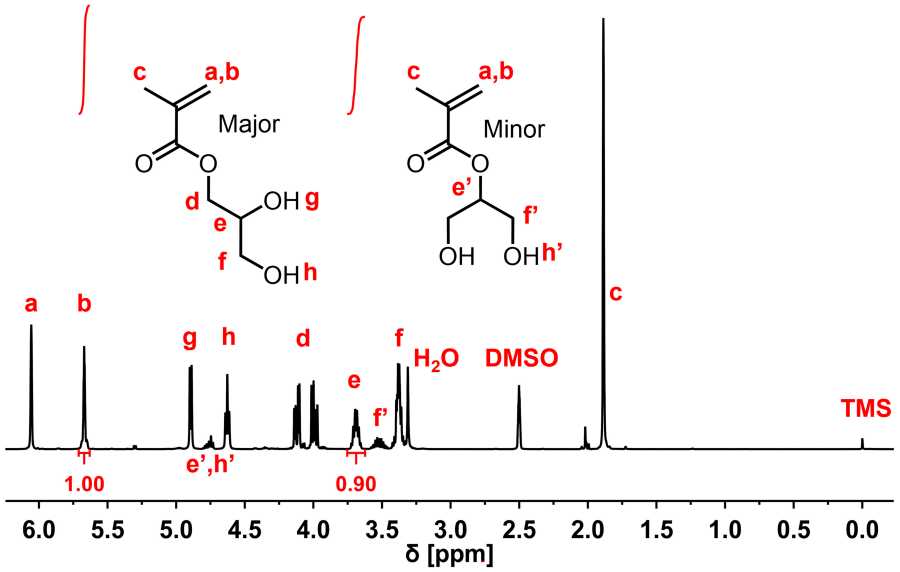


**Figure S2.** $^1H$ NMR spectrum of DHPMA.

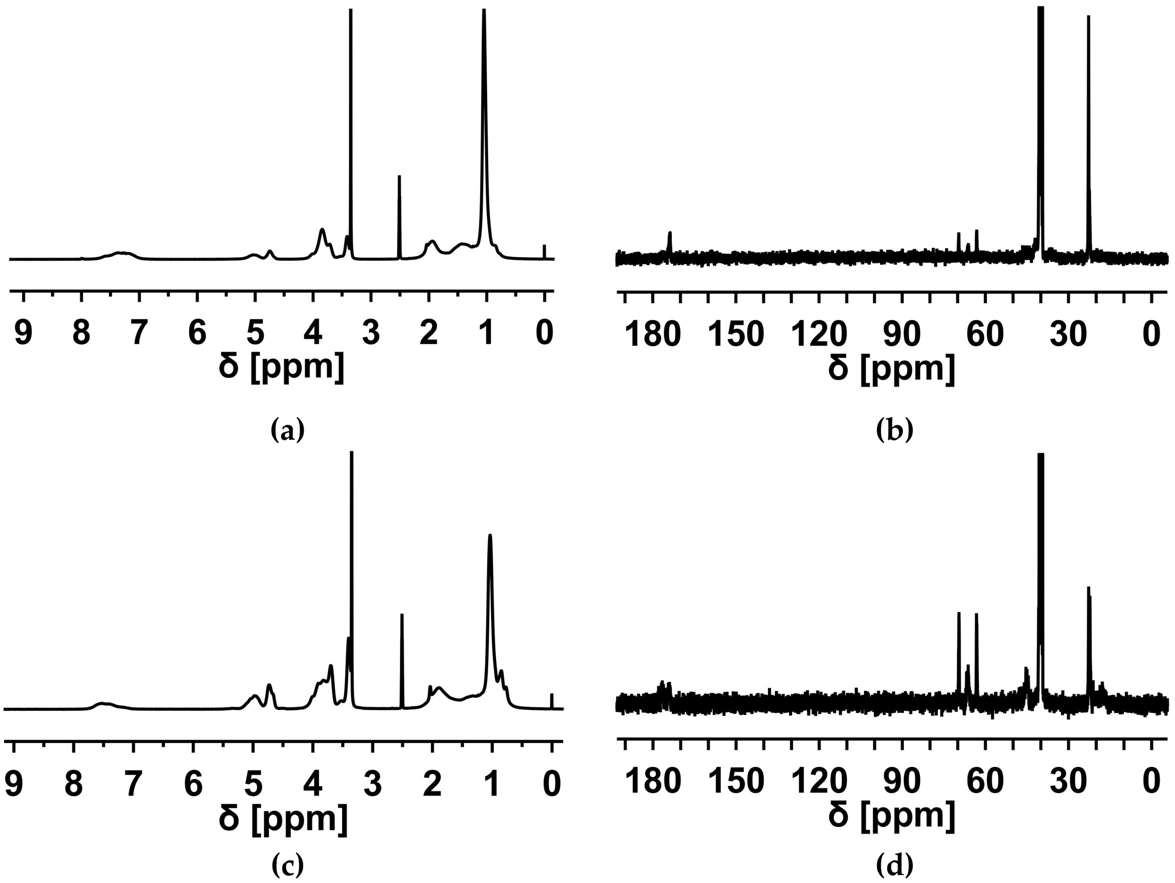


**Figure S3.** $^1H$ NMR and $^{13}C$ NMR of PND 8-2 and PND 6-4 copolymer. PND 8-2: (a) $^1H$ NMR and (b) $^{13}C$ NMR; PND 6-4: (c) $^1H$ NMR and (d) $^{13}C$ NMR.

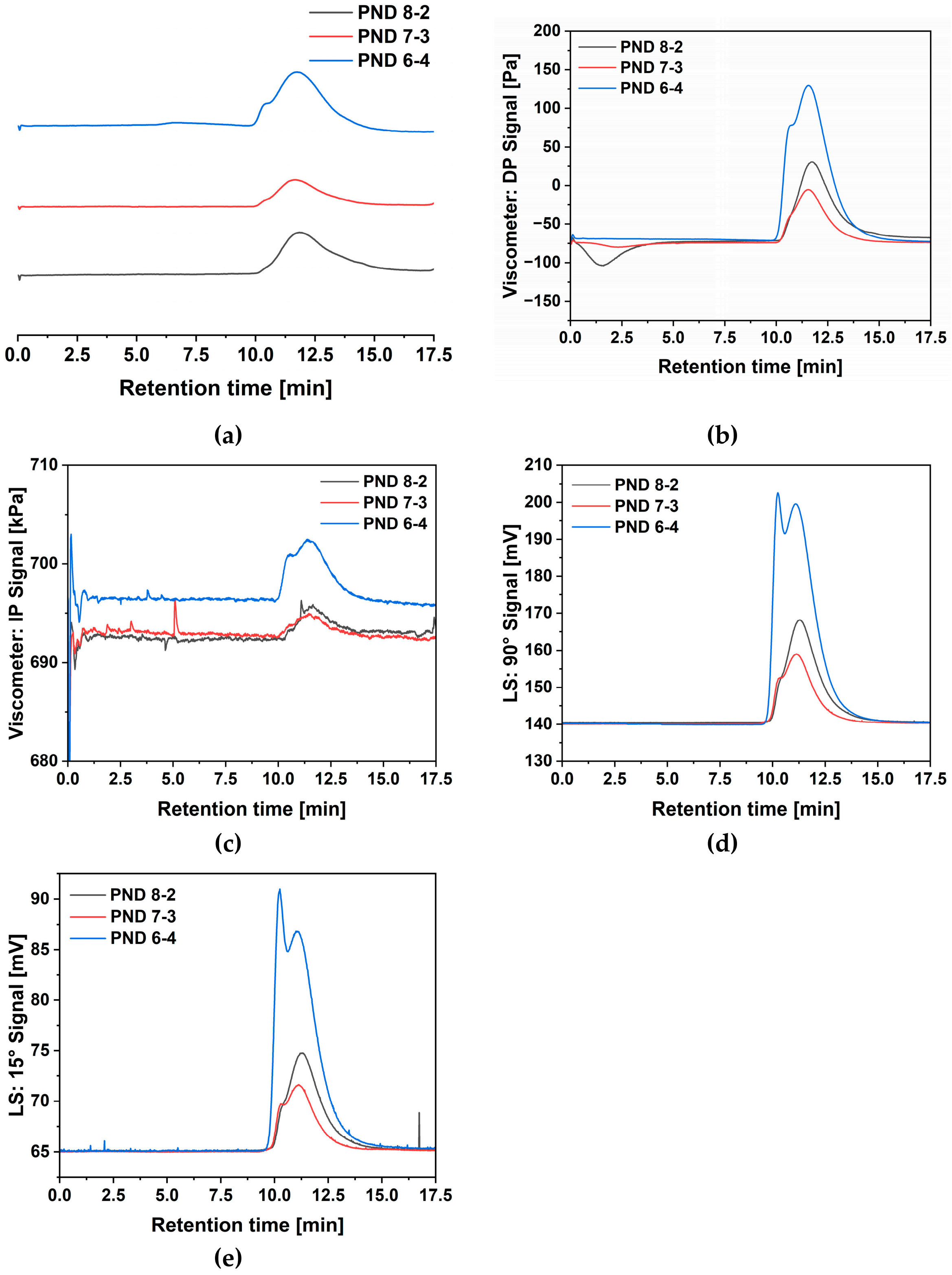


**Figure S4.** GPC results of P(NIPAm-*co*-DHPMA). (a) Differential refractive index detector, (b–c) viscometer and (d–e) light scattering detector.

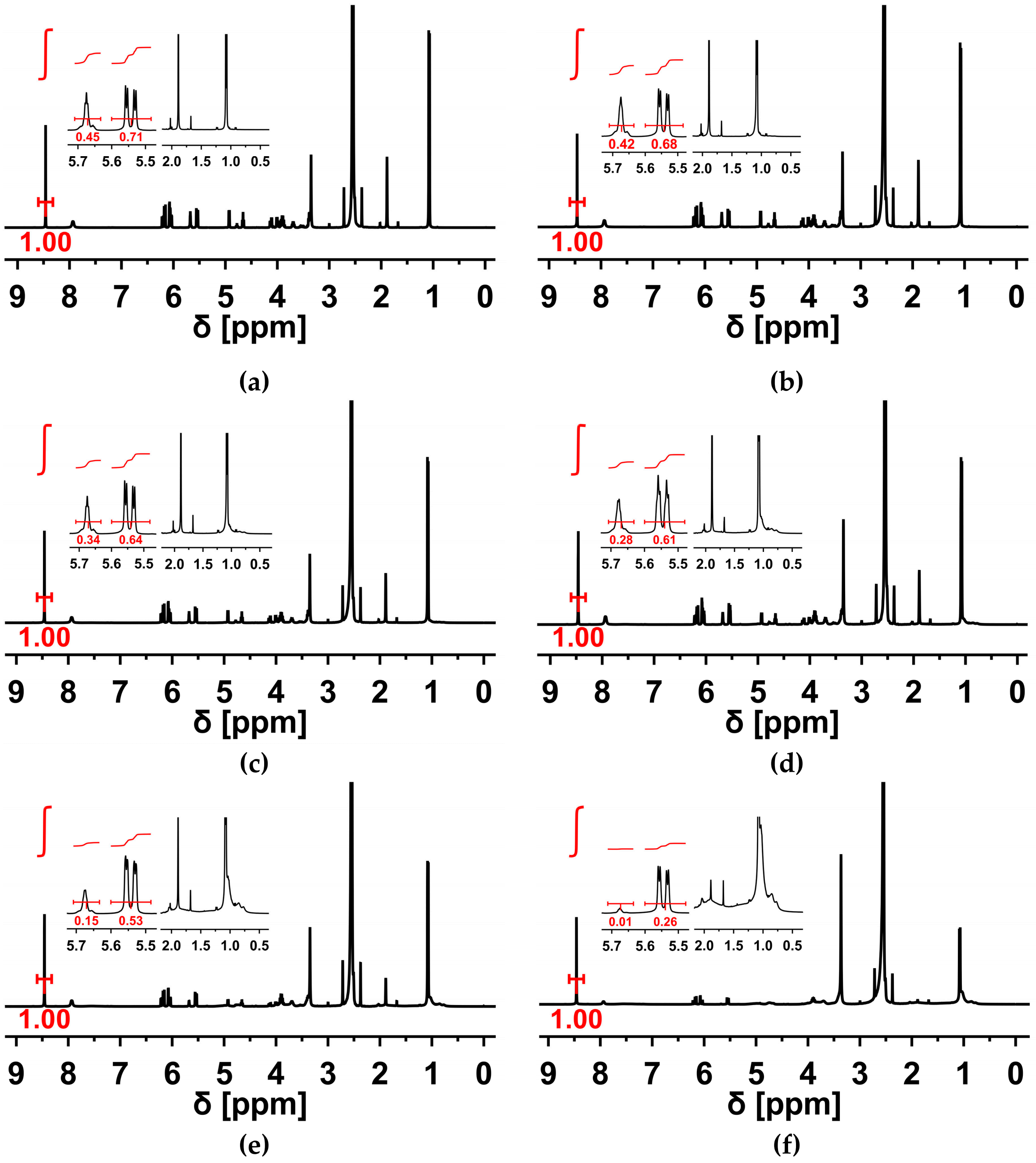


**Figure S5.** $^{1}$H NMR of the conversion of PND 6-4 group at different time. (a) 0 min, (b) 10min, (c) 20min, (d) 30 min, (e) 60min, (f) 120min.

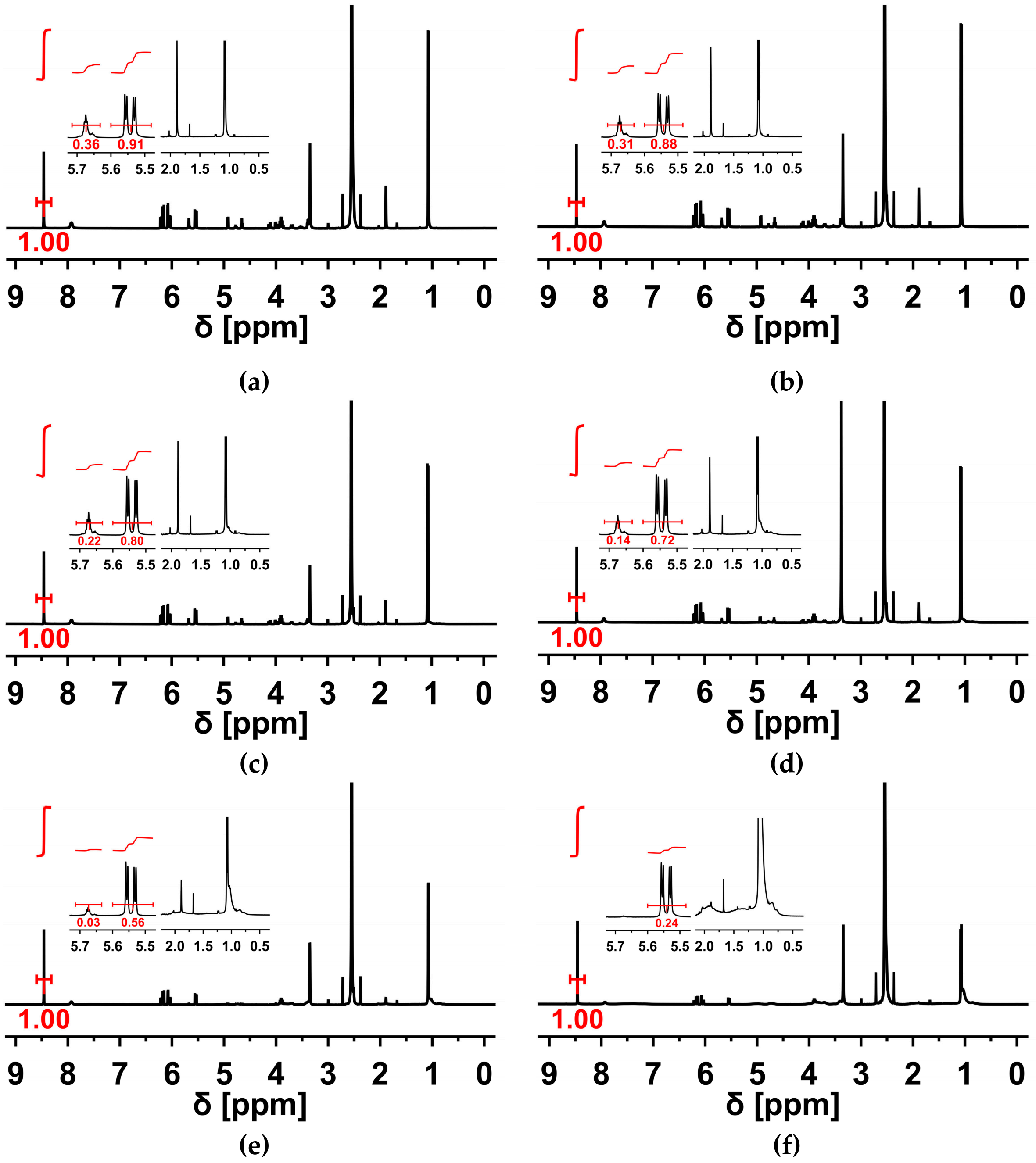


**Figure S6.** $^1$H NMR of the conversion of PND 7-3 group at different time. (a) 0 min, (b) 10min, (c) 20min, (d) 30 min, (e) 60min, (f) 120min.

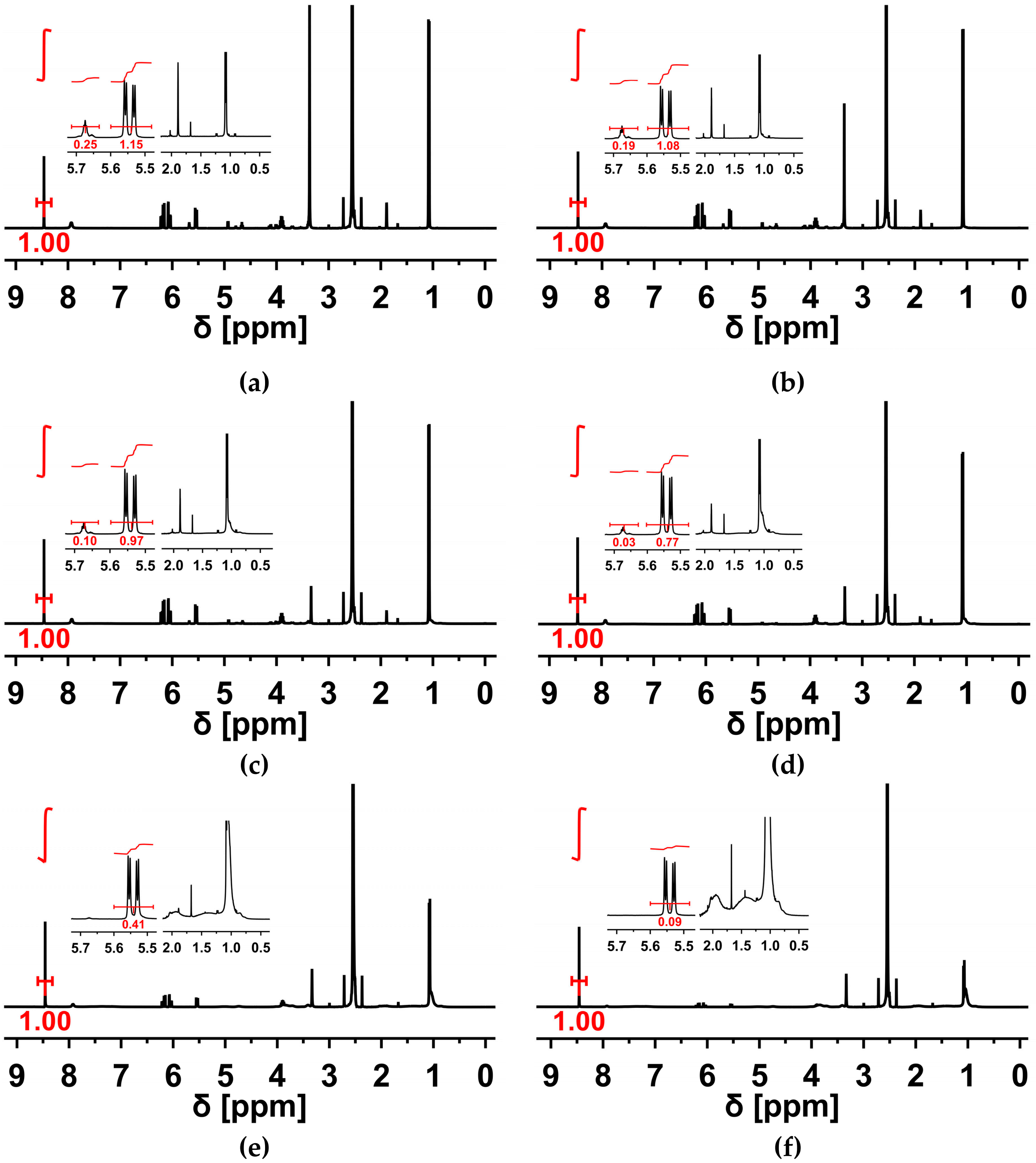


**Figure S7.** $^{1}H$ NMR of the conversion of PND 8-2 group at different time. (a) 0 min, (b) 10min, (c) 20min, (d) 30 min, (e) 60min, (f) 120min.

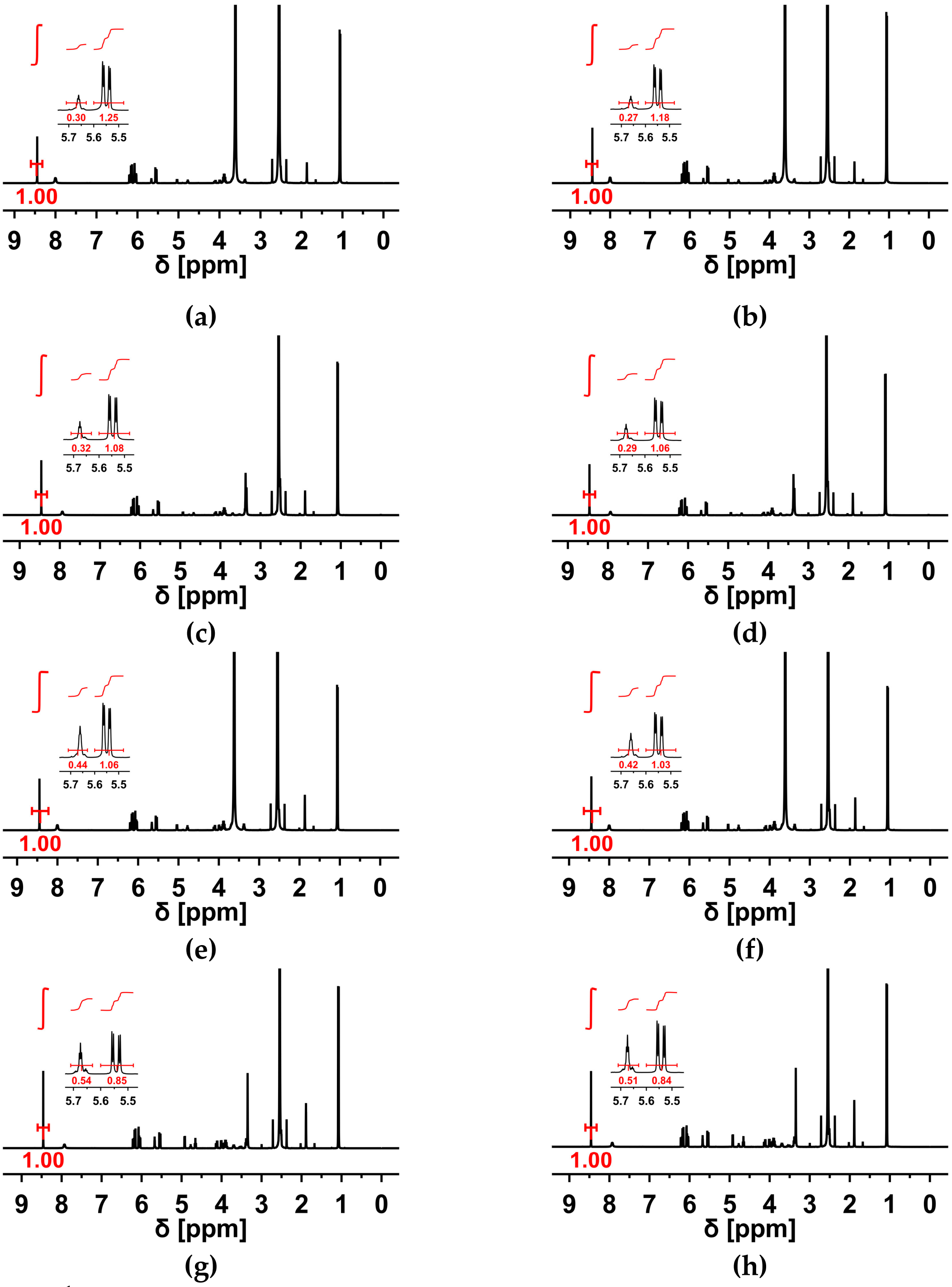


**Figure S8.** $^{1}$H NMR of the conversion of PND 8-2, PND 7.5-2.5, PND 7-3, PND 6-4 group after 8-minute reaction. (a) PND 8-2 0 min, (b) PND 8-2 8 min, (c) PND 7.5-2.5 0 min, (d) PND 7.5-2.5 8 min, (e) PND 7-3 0 min, (f) PND 7-3 8 min, (g) PND 6-4 0 min, (h) PND 6-4 8 min.

**Table S1**. Reaction information for the copolymer.

| Polymer name | Total monomer [mmol] | NIPAm [mmol] | DHPMA [mmol] | AIBN [mol%] | DMSO [mL] | Reaction time [min] |
|---|---|---|---|---|---|---|
| PNIPAm | 33.00 | 33.00 | 0 | 0.50 | 30.00 | 120 |
| PND 8-2 | 33.00 | 26.40 | 6.60 | 0.50 | 30.00 | 120 |
| PND 7.5-2.5 | 33.00 | 24.75 | 8.25 | 0.50 | 30.00 | 120 |
| PND 7-3 | 33.00 | 23.10 | 9.90 | 0.50 | 30.00 | 120 |
| PND 6-4 | 33.00 | 19.80 | 13.20 | 0.50 | 30.00 | 120 |

**Table S2.** Elemental analysis.

| Polymer name | C [%] | H [%] | N [%] | O [%] |
|---|---|---|---|---|
| PND 8-2 | 56.09 | 9.25 | 7.80 | 27.39 |
| PND 7-3 | 56.08 | 8.88 | 5.94 | 29.68 |
| PND 6-4 | 54.71 | 8.50 | 4.37 | 32.52 |
| PND 8-2 8min | 55.46 | 8.49 | 5.00 | 31.19 |
| PND 7.5-2.5 8min | 54.87 | 8.58 | 4.35 | 32.22 |
| PND 7-3 8min | 53.88 | 8.27 | 3.56 | 33.49 |
| PND 6-4 8min | 52.82 | 8.04 | 2.83 | 34.65 |

**Table S3.** Correlation peaks and their attribution in $^1$H-$^1$H COSY spectrum.

| **Correlation peak** | **Attribution** |
|---|---|
| (1.04 ppm, 3.83 ppm) | (–CH–(C$\underline{H}_3$)$_2$, –C$\underline{H}$–(CH$_3$)$_2$) |
| (3.40 ppm, 4.74 ppm) | (–CH–C$\underline{H}_2$–OH, –CH–CH$_2$–O$\underline{H}$) |
| (3.70 ppm, 4.99 ppm) | (–CH$_2$–C$\underline{H}$–OH, –CH$_2$–CH–O$\underline{H}$) |
| (3.40 ppm, 3.70 ppm) | (–CH–C$\underline{H}_2$–OH, –CH$_2$–C$\underline{H}$–OH) |

**Table S4.** Correlation peaks and their attribution in HSQC spectrum.

| **Correlation peak** | **Attribution** |
|---|---|
| (0.85 ppm, 17.52 ppm) | (–C–C$\underline{H}_3$, –C–$\underline{C}$H$_3$) |
| (1.04 ppm, 22.72 ppm) | (–CH–(C$\underline{H}_3$)$_2$, –CH–($\underline{C}$H$_3$)$_2$) |
| (1.40 ppm, 22.45 ppm) | (–CH–C$\underline{H}_2$–, –CH–$\underline{C}$H$_2$–) |
| (1.91 ppm, 41.81 ppm), | (–C$\underline{H}$–CH$_2$–, –$\underline{C}$H–CH$_2$–) |
| (3.40 ppm, 63.11 ppm) | (–CH–C$\underline{H}_2$–OH, –CH–$\underline{C}$H$_2$–OH) |
| (3.54 ppm, 60.04 ppm) | (–CH(C$\underline{H}_2$OH)$_2$, –CH(C$\underline{H}_2$OH)$_2$) |
| (3.70 ppm, 69.56 ppm) | (–CH$_2$–C$\underline{H}$–OH, –CH$_2$–$\underline{C}$H–OH) |
| (3.83 ppm, 40.31 ppm) | (–C$\underline{H}$–(CH$_3$)$_2$, –$\underline{C}$H–(CH$_3$)$_2$) |
| (3.90 ppm, 66.17 ppm) | (–O–C$\underline{H}_2$–, –O–$\underline{C}$H$_2$–) |

**Table S5**. GPC results for P(NIPAm-*co*-DHPMA) with different feeding ratios.

| Polymer name | $M_w$ [g/mol] | $M_n$ [g/mol] | PDI |
|---|---|---|---|
| PND 8-2 | 218860 | 57820 | 3.79 |
| PND 7-3 | 426270 | 234160 | 1.82 |
| PND 6-4 | 362160 | 217880 | 1.66 |

**Table S6**. Information for 8 min copolymerization and the reactivity ratio.

| Name | Initial feeding ratio DHPMA/NIPAm[a] | Conversion [%] | | DHPMA/NIPAm in copolymer[b] | $r_1$ | $r_2$ | $R^2$ |
|---|---|---|---|---|---|---|---|
| | | NIPAm | DHPMA | | | | |
| PND 8-2 | 0.23 | 6% | 10% | 1.12 | 3.09 | 0.11 | 0.99 |
| PND 7.5-2.5 | 0.27 | 2% | 9% | 1.37 | | | |
| PND 7-3 | 0.41 | 3% | 5% | 1.81 | | | |
| PND 6-4 | 0.61 | 1% | 6% | 2.43 | | | |

[a] Initial feeding molar ratios of the monomers were measured by $^1$H NMR.

[b] Ratios between the monomer units in copolymer were calculated from data of elemental analysis.
